# 16S and 18S rDNA Amplicon Sequencing Analysis of Aesthetically Problematic Microbial Mats on the Walls of the Petralona Cave: The Use of Essential Oils as a Cleaning Method

**DOI:** 10.3390/microorganisms11112681

**Published:** 2023-10-31

**Authors:** Natalia Tsouggou, Alexandra Oikonomou, Konstantinos Papadimitriou, Panagiotis N. Skandamis

**Affiliations:** 1 Laboratory of Food Quality Control & Hygiene, Department of Food Science & Human Nutrition, Agricultural University of Athens, Iera Odos 75, 11855 Athens, Greece; n.tsouggou@go.uop.gr (N.T.); pskan@aua.gr (P.N.S.); 2 Ephorate of Palaeoanthropology and Speleology, Hellenic Republic Ministry of Culture and Sports, Ardittou 34b, 11636 Athens, Greece; aloikonomou@culture.gr

**Keywords:** Petralona Cave, EOs, Metagenomics, 16SrDNA, 18SrDNA, functional prediction, *Proteobacteria*, *Actinobacteria*, *Opisthokonta*, SAR

## Abstract

The presence of microbial communities on cave walls and speleothems is an issue that requires attention. Traditional cleaning methods using water, brushes, and steam can spread the infection and cause damage to the cave structures, while chemical agents can lead to the formation of toxic compounds and damage the cave walls. Essential oils (EOs) have shown promising results in disrupting the cell membrane of bacteria and affecting their membrane permeability. In this study, we identified the microorganisms forming unwanted microbial communities on the walls and speleothems of Petralona Cave using 16S and 18S rDNA amplicon sequencing approaches and evaluated the efficacy of EOs in reducing the ATP levels of these ecosystems. The samples exhibited a variety of both prokaryotic and eukaryotic microorganisms, including *Proteobacteria*, *Actinobacteria*, *Bacteroidetes*, *Chloroflexi*, *Firmicutes*, the SAR supergroup, *Opisthokonta*, *Excavata*, *Archaeplastida*, and *Amoebozoa*. These phyla are often found in various habitats, including caves, and contribute to the ecological intricacy of cave ecosystems. In terms of the order and genus taxonomy, the identified biota showed abundances that varied significantly among the samples. Functional predictions were also conducted to estimate the differences in expressed genes among the samples. Oregano EO was found to reduce ATP levels by 87% and 46% for black and green spots, respectively. Consecutive spraying with cinnamon EO further reduced ATP levels, with reductions of 89% for black and 88% for green spots. The application of a mixture solution caused a significant reduction up to 96% in ATP levels of both areas. Our results indicate that EOs could be a promising solution for the treatment of microbial communities on cave walls and speleothems.

## 1. Introduction

Caves can be a source of important historical and biological information that has not yet come to light. Elements related to the prehistoric era have been found, while fossils, paintings, and other artifacts discovered may be important for humanity [1]. From a biological perspective, cave ecosystems are isolated and of great interest as they have followed a unique evolution path and new species can be discovered in them even today.

Every day, show caves attract many tourists [2]. The transformation of caves from precluded environments to tourist attractions requires major modifications of their interior. Some of these modifications are improvements of the already existing natural routes, the construction of paths, platforms, and related infrastructure, as well as the installation of lights [2,3]. However, such modifications may also have a negative impact since they may destabilize the environmental equilibrium that was established in the caves. For example, it has been observed that the entry of visitors and the forthcoming inflow of microorganisms causes alterations in the composition of the microbial environment of the caves [4]. Furthermore, the installation of artificial lighting may lead to the development of photosynthetic microflora around the lamps which is known as “Lampenflora” [5]. The term “Lampenflora,” which means “plants of the lamp” in German, was coined in 1963 by Dobat (1963), and it is now used universally to designate any sort of plant that grows close to lamps [6]. Light wavelength and intensity, temperature, relative humidity, the presence of water, and the type of underlying substrate are some of the factors that may determine the outspread of these phototrophs [7]. The placement of lights in show caves fosters the growth of an intricate community of Lampenflora [5]. The appearance of the cave’s interior may be impacted by the excessive growth of Lampenflora, which has become a widespread issue. In particular, the speleothems, any cave paintings, and generally the substrate on which Lampenflora appears, may be harmed and corroded by the organic acids that may be produced by the related microorganisms [8]. Thus, the inhibition of the growth of Lampenflora, as well as the elimination of the already settled microbial populations is of particular importance. According to studies, these ecosystems mainly consist of *cyanobacteria* and microalgae, such as green algae, diatoms, and mosses [5,6]. Of course, the exact composition of Lampenflora varies between different caves, as well as between different areas inside a single cave. The Lampenflora must be entirely removed from the caves, and this must be carried out in such a way that is harmless for both the health of visitors and the environment of the caves. Since caves are confined spaces, herbicides, which are used in agriculture, should not be applied [6]. Over time, several techniques have been utilized for the refinement of cave walls. Water, brushes, and steam have been used for cleaning speleothems, but they should be avoided. More specifically, using each of these approaches may increase the risk of spreading the contamination throughout the cave [9]. Lampenflora can strongly attach to abiotic surfaces and detach with difficulty, which can cause damage to cave walls and cave structures if not properly treated [1,10]. The growth of photosynthetic organisms can be significantly lowered by reducing the intensity of light and the use of special lamps that emit light at wavelengths different from the spectra of the absorption of Lampenflora [1]. Another option is to use ultraviolet radiation, which has antimicrobial properties, but this is not a permanent solution as suppression of Lampenflora growth may not be sustained [9,10]. In contrast, ozone lamps are not very effective against Lampenflora. It has also been observed that Lampenflora spread is repressed if an alternating mode of lighting is used (i.e., alternating periods of light and darkness aiming at longer periods of darkness) [8]. Sodium hypochlorite (NaOCl) and calcium hypochlorite (Ca(OCl)_2_) are two of the known chemical antimicrobial agents due to their high efficiency and low toxicity [1,8,11]. However, the release of chlorine and the subsequent reactions with many compounds of the cave and the oxidation of Fe^2+^ may lead to the formation of toxic and sometimes carcinogenic compounds and may also alter the cave wall substrates. Hydrogen peroxide (H_2_O_2_) is another effective agent against Lampenflora, but even when used at low concentrations it can damage the cave’s surfaces. On many occasions, there is a need to apply non-polar solvents in order to remove chlorophyll α which is not water soluble and is responsible for the green discoloration of cave walls [1].

Essential oils (EOs) are produced as secondary metabolites and can be obtained through distillation from many plants. They can interact with bacterial cell membranes due to their high hydrophobicity. Thus, cell membrane structures can be disrupted leading to the leakage of molecules from bacterial cells and can even cause the death of the bacteria. Therefore, the potential antibacterial and antifungal properties of these oils are of great importance in many different applications [12]. It was recently established that certain EOs are effective antimicrobials against bacterial and fungal isolates from the Petralona Cave [13]. This cave is a karst formation found on the Chalkidiki peninsula in Greece, 300 m above sea level, one mile east of the settlement of Petralona, and 55 km from Thessaloniki [14,15]. It was discovered in 1959 and when a fossilized archaic human skull, referred to as the “Petralona Skull”, was found there in 1960, the cave gained widespread regard. It quickly drew the attention of geologists and paleontologists due to its remarkable stalactite and stalagmite formations as well as its large collection of fossils. After years of excavations, the cave is now accessible to the public, while nearby there is an archaeological museum where findings from the cave’s interior are exhibited. The cave has a surface area of 10,400 m^2^ and the corridors are around 2000 m long. (http://odysseus.culture.gr/h/2/gh251.jsp?obj_id=1406 (accessed on 3 October 2023)).

The purpose of this study was to identify the microorganisms involved in the formation of problematic microbial ecosystems found in the walls and speleothems of the Petralona Cave using 16S and 18S rDNA amplicon sequencing approaches. We also wanted to test the impact of two different EOs, particularly cinnamon and oregano, on the investigated microflora. Our findings indicate that emulsions of EOs may be efficient against unwanted biofilms formations on cave walls.

## 2. Materials and Methods

### 2.1. Sampling

Samples of unwanted microbial mat found in the Petralona Cave of Chalkidiki were collected from one black and two green spots covering the cave walls. The sampling spots were selected based on the recommendations of the scientific personnel and represented the most severely affected areas found in the cave. This was also corroborated by our own visual assessment since both the green and black spots which were sampled displayed the highest levels of contamination from the unwanted microbial mats within the cave.

Biomass from the surface of the spots was collected using cotton swabs that had previously been immersed in buffered peptone water. In the case of black biofilm, additional scraping was conducted using a sterile scalpel, since the biomass obtained was low. The samples were transferred to the laboratory at 4 °C. Upon arrival, the samples were observed under the microscope with a 100× oil immersion lens to assess the presence/absence of photosynthetic or other microorganisms. Samples were subsequently stored at −20 °C until use. 

### 2.2. DNA Extraction

The microbial biodiversity of the samples from the Petralona Cave was studied using amplicon sequencing. The isolation of the total DNA of the ecosystem of the two green spots, as well as that of the black spot, was achieved using the DNeasy PowerFood Microbial Kit (Qiagen) with slight modifications. Briefly, the cotton was scraped from the swabs with a sterile scalpel so that all biomass was collected in an Eppendorf tube. Then, 500 μL Ringer (half strength) was added and after centrifugation (10,000× *g* for 3 min), 100 μL of lysozyme (50 mg/mL) and 10 μL of lyticase (10,000 U/mL) were added to the pellet and incubated at 37 °C for 30 min. The samples were centrifuged (10,000× *g* for 3 min) and the DNA from the pellet was extracted according to the manufacturer’s instructions, including a step of mechanical lysis with glass beads and rigorous vortexing for 10 min. Eluted DNA was stored at −20 °C. The DNA concentration and the 260/280 and 260/230 nm ratios were measured using a nanodrop device. 16S rDNA was PCR amplified and analyzed by gel electrophoresis to verify the absence of inhibitors that would cause problems during DNA library construction. Specifically, 16S rDNA PCR was performed using the primers: 16S-F 5′-GGAGAGTTAGATCTTGGCTCAG-3′ and 16S-R 5′-AGAAAGGAGGTGATCCAGCC-3′. PCR products were used for gel electrophoresis to assess the quality of the DNA samples.

### 2.3. Sequencing and Bioinformatics Analysis—16S and 18S rDNA Analysis

The microbial ecosystem from the different sites was analyzed by 16S and 18S rDNA amplicon sequencing to characterize the prokaryotic and eukaryotic microorganisms, respectively. Library construction, sequencing, and quality control were performed at Molecular Research DNA (MR DNA, Shallowater, TX, USA). The sequencing reads were imported to the CLC genomics workbench ver. 22 (Qiagen, Hilden, Germany) and the overall analysis was performed as described previously [16]. In brief, chimeric sequence removal and operational unit (OTU) assignment was achieved using the default parameters. For both 16S rDNA and 18S rDNA, genus level OTUs were inferred by using the Silva databases version 132, clustered at 97% identity (Table 1). From all 16S rDNA sequencing datasets, the reads from *Cellulocimicrobium* sp. were excluded from the analysis as contamination from the lyticase reagent used during DNA extraction [17,18].

OTU picking was performed closed to increase the number identified taxa. Alpha-diversity was calculated to assess the adequacy of the sequencing depth of the three samples. Functional analysis of operational taxonomic units (OTUs) of the microbial ecosystems was performed through in silico predictions with PicRust2 [19] and the CLC Microbial Genomics Module’s Infer Functional Profile tool as implemented in the CLC genomic workbench. The functional predictions were based on the EC functional term counts associated with 16S regions in prokaryotes and 18S regions in eukaryotes, using the EC database. The EC numbers were further investigated via heatmap analysis which was performed with normalized clustered Euclidean distances across the samples using default parameters in the CLC genomic workbench.

### 2.4. Application of the Essential Oil Emulsions

Oregano (code:e0070) and cinnamon (code: e0035) EOs were purchased from the PLATON S.A. company. The emulsions applied contained 0.15% oregano oil, 0.4% cinnamon oil, and a mixture of the two oils each in the quantities mentioned above. The main active ingredient in the cinnamon EO solution is Cinnamaldehyde/3-phenyl-propen-2-al. This substance is present in the preparations in the percentage indicated for cinnamon, i.e., 0.4%. In contrast, oregano oil has a carvacrol content of about 70%, which means 0.4% oregano oil corresponds to ca. 0.28% carvacrol. The solutions were prepared in water using polyethylene glycol as an emulsifier. The EOs were applied on the three sampling spots and the viability of the biofilms was assessed with the Charm Firefly2 luminometer, which is appropriate for measuring ATP on various surfaces, reflecting the efficiency of hygiene treatments. Successive spraying of the areas with the three solutions of the EOs was followed. Firstly, the oregano oil was applied for three minutes on the selected area and ATP measurements were performed. After the first spraying, a second one followed with the cinnamon solution. The same procedure was repeated and finally the mixture of the two EOs was applied. ATP concentration was expressed as relative light units (RLU) (1 RLU equals 1 fmol ATP) and the results were compared before and after the application of EOs to evaluate their effectiveness.

### 2.5. Statistical Analysis

The values of the percentage (%) of ATP reduction in the black spot and the green spots were compared using analysis of variance (ANOVA) for *p* < 0.05 followed by the least significant difference (LSD) posthoc test as implemented in Statgraphics XV (Statpoint Technologies Inc., Warrenton, VA, USA).

## 3. Results and Discussion

### 3.1. Observation through Microscopy

**Figure 1:**
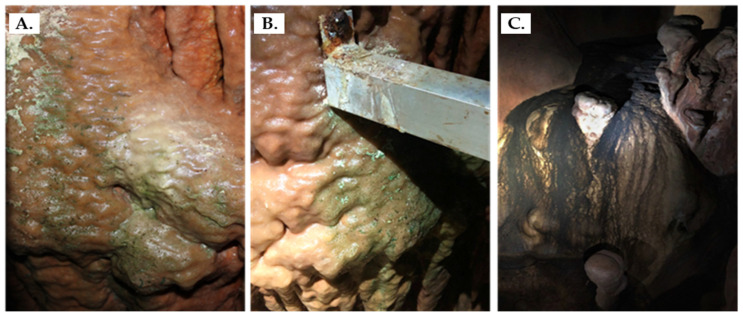
Interior walls of the Petralona Cave where sampling was conducted. The samples were obtained from three distinct spots: Green Spot 1 (**A**), Green Spot 2 (**B**), and the Black Spot (**C**).

The conditions of the cave under which the various treatments were performed were: 83.0 ± 2.0% humidity, 19.5 ± 0.9 °C, 19.00 ± 0.75% O_2_, and 506 ± 16 ppm CO_2_. In the Petralona Cave, the wall microbial communities have spread in many areas, including the cave entrance and deeper regions as well. We were directed by the scientific personnel of the cave to some of the most problematic spots at which biofilm formation was considered to alter the original view of the cave significantly. Two green spots and one black spot were selected for sampling (Figure 1).

**Figure 2:**
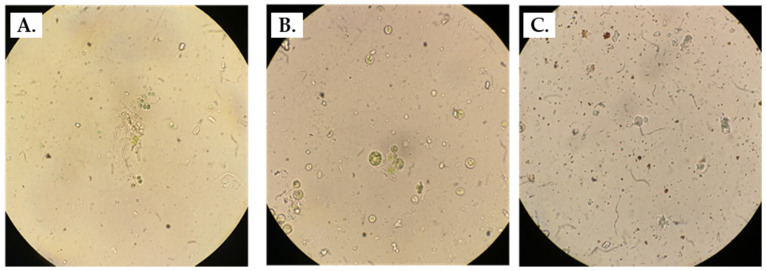
Microscopic examination of the three samples. Observed cell formations of: Green Spot 1 (**A**), Green Spot 2 (**B**), and Black Spot (**C**).

Under the microscope, the samples appeared heterogeneous, and a variety of microbial cell morphologies were visible. Coccoid and bacillary forms were some of the distinctive cell shapes which could potentially belong to bacterial cells. Within both green and black spots, large spheres with irregular surfaces were also occasionally seen. The samples derived from green Lampenflora mainly consisted of green microorganisms although in some cases brown–red cell formations were also spotted. The morphology of the organisms does not allow us to distinguish whether they are algae or *Cyanobacteria*. Greenish microorganisms were also detected in the black spots. Some brown–red cell formations were observed as well (Figure 2).

### 3.2. Description of the Biodiversity of Prokaryotic Microorganisms in the Petralona Cave Biofilms

**Figure 3:**
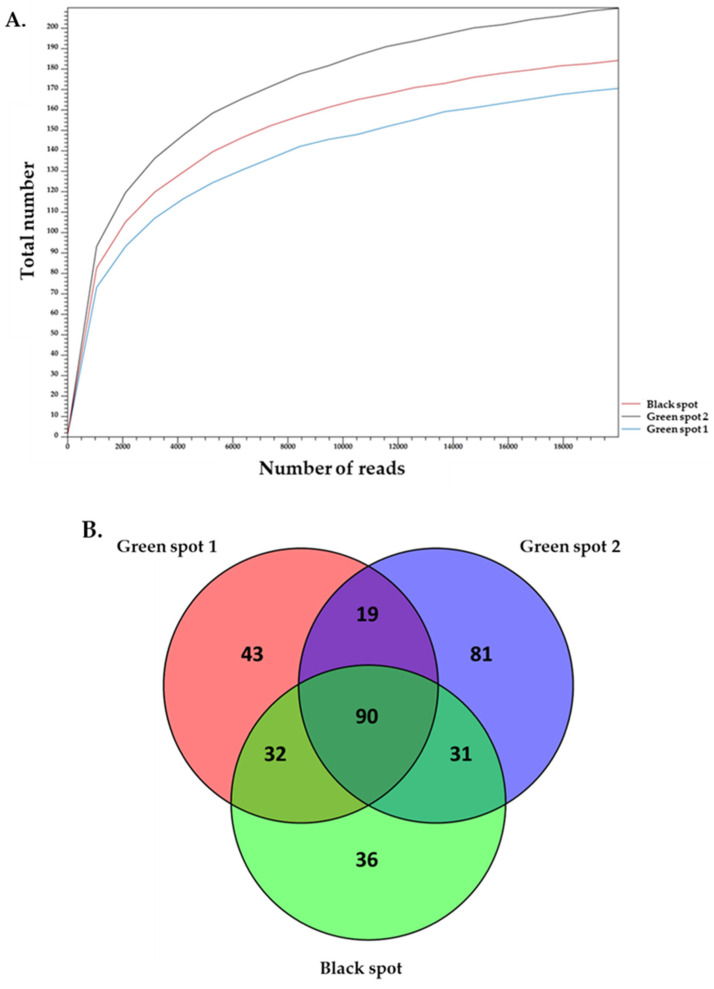
Alpha-diversity analysis of 16S rDNA reads for a maximum depth of 20,000 read counts using the total number of OTUs at genus level (**A**). Venn diagram illustrating the common and distinct genera among the samples Green Spot 1, Green Spot 2, and the Black Spot. Each sample is represented by a separate circle, and their intersections show the shared genera among them (**B**).

The 16S rDNA amplicon sequencing results were analyzed to identify the prokaryotic microorganisms that make up the ecosystems of the three samples. Alpha-diversity analysis showed that the sequence depth was adequate for the three samples. It also demonstrated that Green Spot 2 had a greater complexity than both Green Spot 1 and the Black Spot (Figure 3A).

**Figure 4:**
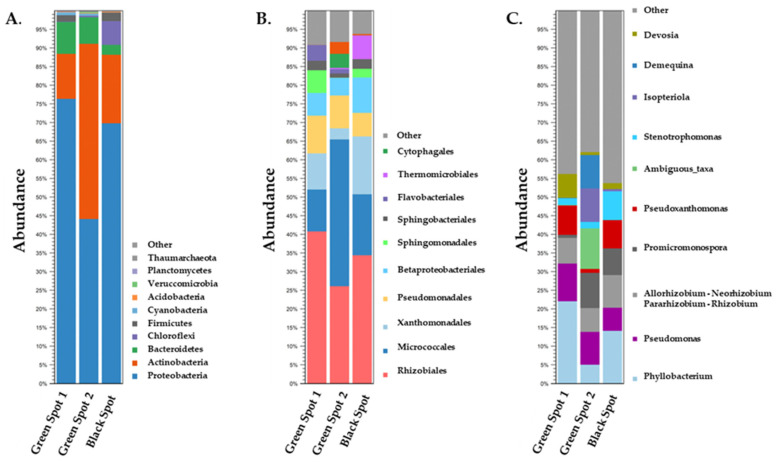
Taxonomic profile and relative abundance of the three samples based on the 16S rDNA amplicon data at phylum (**A**), order (**B**), and genus levels (**C**).

At the kingdom level, the dominant prokaryotic microorganisms across all three samples were identified as *Bacteria* (>99% abundance), with only a very small proportion belonging to *Archaea*. The clustering of 16S rDNA reads of the three samples based on phylum, order, and genus levels is shown in Figure 4. As it is evident there is a rich biodiversity in the samples while it also appears that there are some common microorganisms found among them.

The predominant phyla were *Proteobacteria* ranging between 44% and 76% apart from Green Spot 2, in which *Actinobacteria* appeared with the highest abundance of 47%. In the other two samples *Actinobacteria* were the second largest population with abundance ranging from 12 to 18%. Overall, *Proteobacteria* have previously been described in studies on cave biota. More specifically, *Proteobacteria* dominated in the investigated biofilms found in the Wind Cave in the USA [20], in the limestone microbial mats in the Frassasi Cave in Italy [21], and on the walls of the Yumugi River Cave in New Guinea [22]. *Proteobacteria* are known for their capacity to degrade a variety of organic substrates and this may partly explain the fact that they can prevail even in caves where the conditions such as pH, temperature, and nutritional stress are not expected to be favorable for their growth [23]. *Actinobacteria* are also very common in caves [24,25,26]. Cave-dwelling *Actinobacteria* are unique because they live in extreme and often pristine environments, which can result in the exploitation of various metabolic pathways, including biomineralization, rock-weathering [27], and biodegradation [28]. *Actinobacteria* are capable of scavenging trace gases from the atmosphere, such as H_2_ and CO. This ability allows them to contribute to the primary productivity, colonization, and development of microbial life in nutrient-poor habitats (i.e., oligotrophic cave environments) [29]. *Bacteroidetes* constituted the third most abundant phylum in both Green Spot 1 and Green Spot 2, accounting for 9% and 7% of the microbial population, respectively. In the Black Spot, *Chloroflexi* was the third most prevalent phylum, comprising 6% of the total population. *Firmicutes* followed, representing 2% of the microbial community in both Green Spot 1 and the Black Spot, but showing a much lower percentage in Green Spot 2 (<1%). The phyla *Bacteroidetes*, *Chloroflexi*, and *Firmicutes* are frequently identified in cave ecosystems and have significant ecological roles [2,30]. *Bacteroidetes* are recognized for their capacity to break down complex organic compounds and therefore may serve as key decomposers in cave environments [31]. *Chloroflexi*, being photosynthetic bacteria that utilize light to produce energy [32] could be detected in cave ecosystems where there is some degree of light penetration. Additionally, *Chloroflexi* have been identified as heterotrophic oligotrophs, with a notable ability to metabolize plant polymers and adapt to nutrient-limited oligotrophic conditions [33]. Previous research has emphasized the crucial role of these bacterial groups in carbon and nutrient cycling in aquatic ecosystems [34]. In addition, the two green spot samples contained photosynthetic *Cyanobacteria*, but with limited abundance (ca. 1% in both spots). Other phyla found in the samples were *Acidobacteria*, *Verrucomicrobia*, *Planctomycetes*, and *Thaumarchaeota* which are all common in caves and soils [35]. 

The present research reveals that the predominant classes were *Alpha-Proteobacteria* and *Gamma-Proteobacteria*, which accounted for a range of 27.2% to 48.6% and 16.7% to 31.3%, respectively, in the three samples. *Alpha-Proteobacteria* and *Gamma-Proteobacteria* are two diverse classes found in many terrestrial and marine environments, including caves [36], and contain genera that are photrophic, plant symbionts and some can be bioluminescent [37]. These two classes were found to be the main representatives of *Proteobacteria* in many studies conducted in caves such as the Iron Curtain Cave in Canada [38], the Spider Cave, and the Lechuguilla Cave in New Mexico [39] and various caves of Mizoram in Northeast India [40]. *Actinobacteria* was the most frequent class of *Actinobacteria* phylum in all the samples appearing with up to 45.6% abundance in the Green Spot 2.

*Rhizobiales* were the most prevalent order of Green Spot 1 and the Black Spot, representing 40.9% and 34.7% of the microbial population, respectively. In both Green Spot 1 and the Black Spot, *Micrococcales* were the second most frequent order in the bacterial communities, with 11.1% and 16% abundance, respectively. However, at Green Spot 2, *Micrococcales* was the most prevalent order, representing 39.2% of the microbial population, while *Rhizobiales* appeared second with 26.1% abundance. Several new species of *Rhizobiales* have been identified in caves, where they thrive in underground habitats [22,41]. These species are recognized for their ability to perform nitrogen fixation and for their significant involvement in the breakdown of organic materials [42,43]. In a study conducted in a cave in north-western Romania, *Micrococcales* were among the members of the analyzed bacterial communities [44]. In another study, *Micrococcales* were also identified as one of the bacterial orders existing in the Yumugi River Cave [22]. Members of the *Xanthomonadales* and *Pseudomonadales* were classified as the next two richest orders with the first reaching a 15.4% abundance in the Black Spot and the second 10.1% and 8.7% in Green Spot 1 and Green Spot 2, respectively. *Xanthomonadales* were previously detected in various caves, including a karstic cave in Slovenia [23], a cave in north-western Romania [44], and in the yellow colonizations present on the walls of the Altamira Cave in Spain [26]. In the same caves investigated by Bogdan et al. and Portillo et al., the order *Pseudomonadales* was also present in the sediments and wall samples. Two additional notable orders, *Betaproteobacteriales* and *Sphingomonadales* were also detected. *Betaproteobacteriales* was at its highest abundance in the Black Spot at 9.5%, while *Sphingomonadales* was at its highest abundance in the Green Spot 1 (5.8%). *Betaproteobacteriales* is a diverse order of bacteria that have frequently been detected in various environments, including caves [25,29]. Some *Betaproteobacteriales* members are involved in nitrogen cycling, while others are involved in sulfur cycling [45,46]. *Sphingomonadales* have also been found to colonize caves. They are capable of utilizing different organic compounds as energy and nutrient sources, which allows them to survive and grow in such environments [44,47]. Furthermore, bacteria of the orders *Sphingobacteriales*, *Thermomicrobiales*, and *Cytophagales* were also present in our samples but with lower abundances. Of note, *Thermomicrobiales* were identified in a significant amount in the Black Spot, with 6% abundance. This order has previously been identified in samples obtained from a fresco painting inside the Cave Church of the Sts. Peter and Paul [48]. In addition, it has been observed that bacteria of this order, which are capable of withstanding stressful conditions, have demonstrated improved functional pathways related to the metabolism of amino acids and carbohydrates [49]. These metabolic pathways have been reported to act as agents of osmotic adjustment, allowing the bacteria to cope better with severe drought stresses. 

At the genus level, multiple genera were identified in the samples with varying abundances. *Phyllobacterium* was the most common genus, with the highest percentages in Green Spot 1 (21.8%) and the Black Spot (14.3%), but with a lower percentage in Green Spot 2 (5%). In Green Spot 2, bacteria from the *Devosiaceae* family, assigned as ambiguous taxa, were the most abundant (13.6%), followed by *Isoptericola* (11.5%), and *Demequina* (11.2%). The abundance of these genera was very low in the other two samples. *Promicromonospora* had a higher abundance in Green Spot 2 and the Black Spot, with 9.4% and 7%, respectively, while in Green Spot 1, it appeared with only <1% abundance. *Pseudomonas* and members of the *Rhizobium*/*Agrobacterium* group were present in all samples (ranging from 6.1% to 10% and 6.4% to 8.8%, respectively). Other notable genera identified in all three samples, but with varying abundance levels, included *Pseudoxanthomonas*, *Stenotrophomonas*, and *Devosia*. Previous studies conducted in caves are in agreement with our results regarding the presence of all the genera of *Proteobacteria* that we have described above [50]. *Phyllobacteria* are typically associated with plants and are commonly found in root nodules [51] and the rhizosphere [52], but they have also been discovered in subterranean environments such as Roman catacombs [53] and caves like Lascaux [54], Ardales [55], and El Castillo [56]. *Phyllobacteria* are also present as free-living bacteria in both soil and water [57], indicating their adaptive capacity to thrive in diverse environments. In addition, Duncan et al., who investigated the microbial populations from the nutrient-limited Lechuguilla and Spider caves, revealed that *Pseudomonas*, *Stenotrophomonas*, and *Pseudoxanthomonas* were part of the investigated ecosystems [39]. *Pseudomonas* are ubiquitous bacteria that are involved in organic matter degradation and have been found in various environments such as water, soil, and plants [58]. In addition to *Pseudomonas*, other Gram-negative bacteria such as *Stenotrophomonas* and *Rhizobium* are commonly associated with *cyanobacterial* filaments and nitrogen assimilation in underground environments and caves [59,60]. *Devosia* have been also detected in various caves, including the Iron Curtain Cave in Canada and caves in the mountains of the Hindu Kush [38,61]. Moreover, previous studies have also reported the presence of *Promicromonospora* and *Isoptericola* in cave environments. For example, *Promicromonospora* were isolated from the Hampoeil Cave [62] and from the environment of the Zuheros Cave in Córdoba [63]. *Isoptericola* was found in St. Agatha’s Catacombs in Rabat in Malta [63] and in the Sigangli Caves in China [28]. *Isoptericola* species have been found to exhibit mineral-weathering and chitin-degrading capabilities [64,65], which could be an important source of nutrients for other organisms in cave ecosystems. Although the presence of Demequina in caves has not been studied extensively, the genus has been identified in many environmental habitats, including soil, sediment, and water [66].

### 3.3. Description of the Biodiversity of Eukaryotic Microorganisms in the Petralona Cave Biofilms

**Figure 5:**
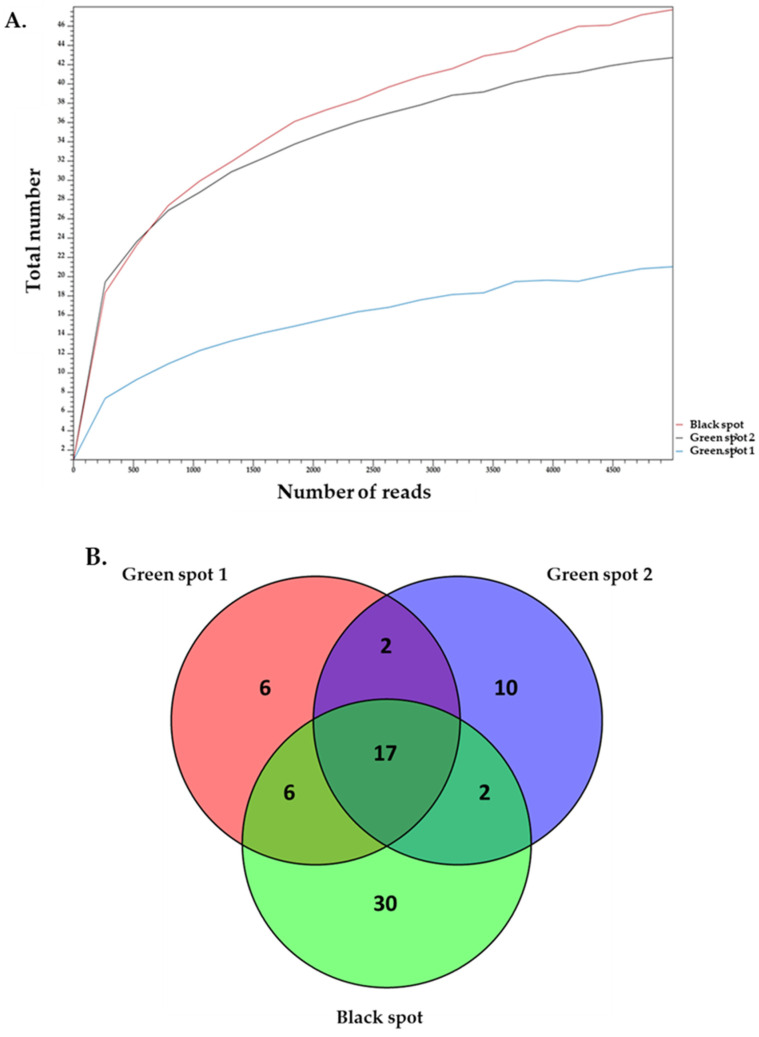
Alpha-diversity analysis of 18S rDNA reads for a maximum depth of 20,000 read counts using the total number of OTUs at genus level (**A**). Venn diagram illustrating the common and distinct genera among the samples Green Spot 1, Green Spot 2, and Black Spot. Each sample is represented by a separate circle, and their intersections show the shared genera among them (**B**).

In addition to a diverse assortment of bacteria, the samples exhibited a variety of eukaryotic microorganisms. The 18S rDNA amplicon sequencing results were analyzed to identify the eukaryotic microorganisms that participate in the formation of the ecosystems of the three samples. Alpha-diversity confirmed that the sequence depth was adequate for all three samples, and revealed that the Black Spot had a greater complexity than the other two ecosystems (Figure 5).

**Figure 6:**
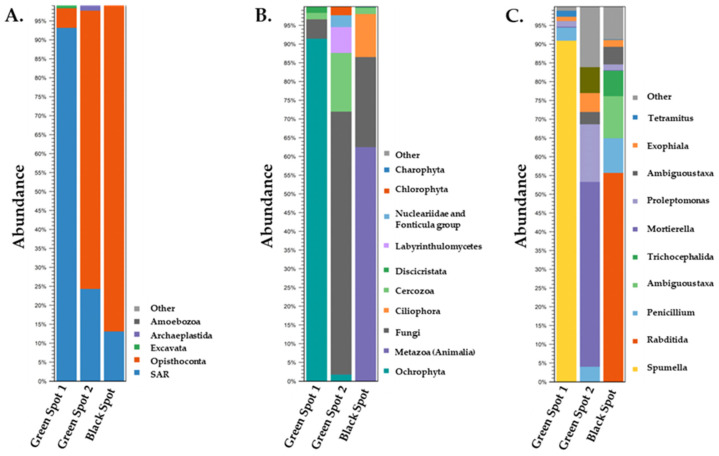
Taxonomic profile and relative abundance of the three samples based on the 18S rDNA amplicon data at phylum (**A**), order (**B**), and genus levels (**C**).

Upon conducting primary analysis of the samples at the kingdom level, it was observed that the greatest proportion of eukaryotic organisms detected in the samples were unspecified microorganisms. This is a frequent occurrence since environmental samples exhibit a vast array of biodiversity, and numerous microorganisms from such ecosystems cannot be cultured in a laboratory, therefore remaining unidentified. Consequently, we only proceeded with analyzing eukaryotic organisms that could be identified which all belonged to *Eukaryota* (Figure 6).

At phylum level, in the Green Spot 1 sample, the SAR supergroup, consisting of *Stramenopila*, *Alveolata*, and *Rhizaria*, clearly dominated (93%). The SAR supergroup was also present in the other two samples but in decreased abundance, specifically, 24% in Green Spot 2 and 13% in the Black Spot. This supergroup includes a variety of organisms such as diatoms, algae, ciliates [67], and foraminifera [68], and is known to thrive in cave ecosystems [69,70]. In both the Green Spot 2 and Black Spot samples *Opisthokonta* dominated with high percentages (>73% abundance). In Green Spot 1, *Opisthokonta* is also present, but it accounted for just 5% of the sample. *Opisthokonta* is a taxonomic supergroup that includes various eukaryotic organisms, like fungi, animals, and some protists [71]. Many species of opisthokonts are found in caves [30,69], where they can exhibit important ecological roles as decomposers [72], predators [73], and symbionts [74]. Overall, *Opisthokonta* are significant contributors to the biodiversity and ecological intricacy of cave ecosystems. Other phyla present in the samples include *Excavata*, *Archaeplastida*, and *Amoebozoa*. These phyla are distributed with different percentages among the three samples and are often found in various habitats, including caves [70]. *Excavata* is a diverse group of unicellular organisms that includes parasites [75] heterotrophic predators and photosynthetic members [68,76]. *Archaeplastida* consists of eukaryotic organisms that include green algae, red algae, and land plants [77]. Although most members of *Archaeplastida* are not typically found in caves, there are some studies which had previously reported their presence in such environments [78]. *Amoebozoa* is a group consisting of various species of both free-living amoebae and parasites [79]. Among these species, some are known to inhabit cave systems and play significant ecological roles [80]. 

In terms of the order taxonomy, *Ochrophyta*, *Metazoa*, and *Fungi* were some of the most prominent groups with abundances that varied a lot among the samples. Specifically, *Ochrophyta* accounted for 91.1% and 1.8% of the total samples in Green Spot 1 and Green Spot 2, respectively. *Ochrophyta*, are a diverse group of photosynthetic organisms that include diatoms, brown algae, and some golden algae [81]. While they are typically associated with aquatic environments, [82] some species have been also found in caves [83,84]. In addition, researchers have previously described *Ochrophyta* as part of the cave Lampenflora, growing close to artificial lighting [85]. In the Black Spot, *Metazoa* appeared to be the most frequently observed order occupying 62.6% of the community. Furthermore, *Ciliophora* were also identified in a significant amount in the Black Spot, accounting for 11.3%, while this order had a very low presence in Green Spots 1 and 2 (<1%). Caves are known to harbor a diverse range of *Metazoa*, including obligate cave dwellers and edaphic species [86] and consist of many bacteria-feeding members [87]. *Ciliophora* are eukaryotic, often parasitic organisms which have been also identified in caves [88]. In Green Spot 2, *Fungi* were the most abundant order, reaching 70%, followed by *Cercozoa* at 15.4%. *Fungi* were also present in Green Spot 1 and the Black Spot, but with lower percentages, specifically 5% and 24.4%, respectively. *Cercozoa* also appeared in low populations in Green Spot 1 (1.9% abundance) and in the Black Spot (1.7% abundance). *Fungi* are particularly abundant in caves, where they can break down organic matter and recycle nutrients in the nutrient-poor environment. Some species of fungi form mutualistic relationships with other organisms (lichens), such as fungi, algae, or cyanobacteria [74]. *Cercozoa* are commonly found in soil and aquatic environments [89]. Although there is limited research on their occurrence in cave ecosystems, some studies have suggested that *Cercozoa* may be present in such habitats [70]. *Cercozoa* are known to be important predators of bacteria and can participate in the regulation of microbial communities in caves [90]. In Green Spot 2 *Labyrinthulomycetes* (6.8%), members of *Nucleariidae* and *Fonticula* (2.9%) group and *Chlrorophyta* (2%) were also found in notable abundances. *Discicristata* is another order which was detected in Green Spot 1 (2%) and the Black Spot. Previous studies have also reported the presence of *Labyrinthulomycetes* [69] and *Chlrorophyta* [91] in caves. In particular, *Chlorophyta*, which consist of photosynthetic members, have been characterized as part of cave Lampenflora [92].

The microbial communities of the three cave spots (Green Spot 1, Green Spot 2, and the Black Spot) showed significant differences at the genus level. In the Green Spot 1, the genus *Spumella*, which is an alga, seemed to be clearly dominant occupying 90.2% of the total sample. *Spumella* has previously been isolated from Lampenflora in Carlsbad Cavern in New Mexico [85] and from ice obtained from the wall areas of the Scarisoara ice cave located in Romania [93]. This genus consists of non-photosynthetic unicellular flagellates with some species being bacterivores [94]. *Mortierella* (49.0%), a genus of fungi known for its ability to degrade plant litter and produce compounds beneficial for microorganism growth [95], was the most abundant genus in Green Spot 2, along with members of the genus *Proleptomonas* (15.0%), a flagellated protozoa typically found in soil [96]. The presence of *Mortierella* members has been reported in the sediments of the Castañar Cave, in Spain [97], and other cave systems [2]. However, we could not find reports about the presence of *Proleptomonas* in caves and its ecological role in such habitats. In the Black Spot, the genus *Rhabditida* predominated with 55.7%, followed by members of the *Colpodida* class with 10.9%, which could not be classified at genus level and is thus referred to as ambiguous taxa. Another important genus which appeared in notable abundances is *Penicillium* (9.4% in the Black Spot, 3.8% in Green Spot 2, and 3.4% in the Green Spot 1). Additional genera were also identified such as *Trichocephalida*, *Exophiala*, and *Tetramitus*, but with decreasing abundances. *Rhabditida* is a group of nematoda that includes both free-living and parasitic species [98] and have been found to inhabit caves [86]. Members of *Rhabditida* are often found living in close association with other organisms, such as insects, plants, and fungi [87]. *Rhabditophanesschneideri*, a species of nematode belonging to *Rhabditida*, was discovered to be phoretic on a cave-dwelling pseudoscorpion species as a means of survival [98]. Members of *Colpodida* can be found in various environments, including soil [99] and also in caves as in the case of the Frasassi Cave in Italy [100]. *Colpodida* thrives in environments with high levels of bacteria, as some of them are bacterivorous [101]. *Penicillium* is a genus of fungi that includes several species that are commonly found in caves. Its presence has been identified in the Cave of Bats, in Spain [60], and also in the Petralona Cave in a previous study [13].

### 3.4. Functional Analysis Using PICRUST2

**Figure 7:**
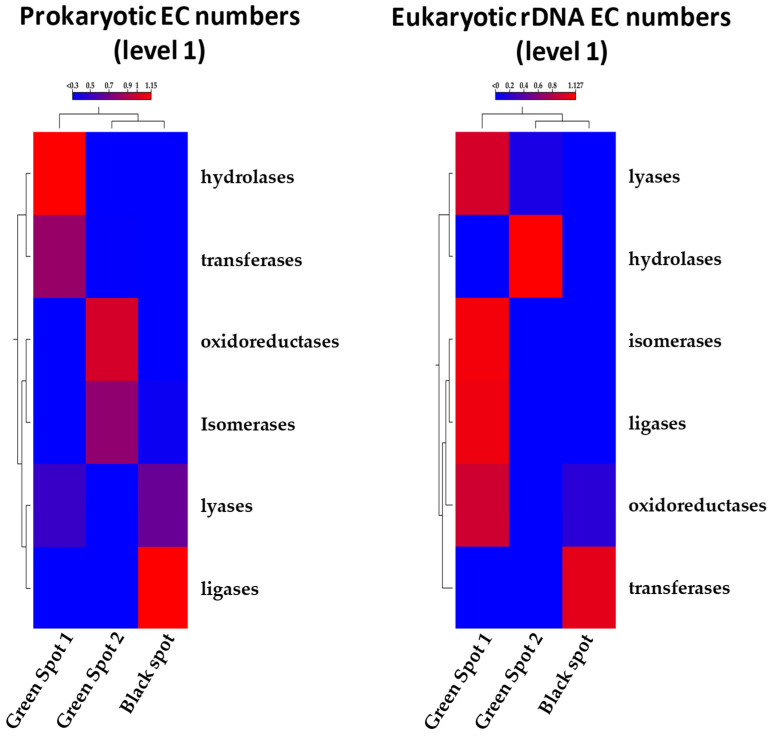
Heatmap showing the relative abundances of predicted enzyme classes by PICRUSt2 across the three different sampling points.

The functional profiles of the collected samples’ microbial communities were predicted by utilizing PICRUSt2 [19] based on the 16S and 18S rRNA sequences. By employing the Enzyme Commission (EC) database, the analysis revealed the presence of five major functional enzymatic categories (EC level 1) in both prokaryotic and eukaryotic ecosystems, namely oxidoreductases, transferases, hydrolases, lyases, and ligases. Heatmaps were constructed for both the 16S and 18S sequences, demonstrating that the analysis at EC level 1 supported that Green Spot 1 and 2 were more similar, while the Black Spot was more distinct. The observed similarity between the green spots may indicate the presence of common prevailing species, influencing the gene content within the respective metagenomes. However, all three samples displayed the presence of the five enzyme categories, but with varying abundances (Figure 7). Further analysis was conducted on the EC numbers at level 2, which enabled the identification of sub-subclasses providing a higher degree of specificity for their functions. The results showed a greater complexity regarding the grouping of the samples based on their functional profiles (Supplementary Figure S1). Consequently, additional research is necessary to investigate the specific functions of the identified enzymes and gain a deeper understanding of the biological roles and interactions of the species involved in the formation of cave ecosystems.

### 3.5. Antimicrobial Action of Essential Oils against the Petralona Cave Biofilms

Certain commercial EO emulsions were applied on the surfaces of the three spots and ATP measurements were recorded (Table 2). It appears that the treatment of the speleothems with EOs was effective. In all cases, after the application of the emulsions for 3 min the percentage of ATP of the microbial communities was reduced on the tested surfaces. More specifically, the treatment with oregano EO was able to reduce the ATP levels by 44–86% and 77% when applied on the green and the black biofilms, respectively. Consecutive spraying with cinnamon ΕO resulted in further reduction in the ATP levels. In particular, when sprayed on the green spots the ATP reduction increased to 88–93% for the green spots and 89% for black area. Finally, the direct application of the mixture of both solutions caused a significant reduction of ≥95% in the measured ATP levels of both areas.

The application of control systems for cultural heritage assets involves continuous monitoring, diagnosis of biodeteriogens, and treatments. Green restoration has gained attention for its use of environmentally friendly and safe substances, with EOs being considered an alternative to synthetic biocides due to their natural and non-toxic properties. As described, EOs seem to be a promising solution for cultural heritage preservation, focusing on their antimicrobial properties [102].

EOs seem to be a promising solution for dealing with the problematic flora which may appear on the walls and speleothems of caves. EOs have shown potential in damaging bacteria through various mechanisms. They can react with the peptidoglycan of Gram-positive bacteria, damaging their membrane [103]. In addition, studies indicate that EOs can disrupt the regulation of potassium transport in bacteria, leading to excessive potassium leakage and cell death [12]. They also affect cell membrane permeability, causing the leakage of carboxyfluorescein loaded cells, suppressing bacterial growth [104,105]. Thymol and carvacrol, two EO components, have been found to increase the intake of N-phenyl-L-naphtylamine (NPN), suggesting the presence of large membrane pores [106]. Maintaining a pH gradient is vital for cell survival. EOs have been shown to alter the internal pH of bacteria, with oregano, savory, cinnamon, bergamot, orange, and vanillin oils affecting the pH gradient of various bacterial strains [107,108,109,110]. 

Another study aimed at preserving oil paintings while addressing the challenge of direct contact between EOs and surfaces, which can result in unexpected reactions with pigments and potential solvent effects. The study focused on testing oregano and clove EOs, known for their inhibitory properties, with a special emphasis on their volatile components. The findings indicated that particularly the volatile elements in oregano EO, successfully prevented the growth of potential biodeteriogens [111].

Regarding the antimicrobial potential of the EOs tested in our study, the application of the solutions on Lampenflora seems to be successful. After applying the oils for 3 min, they all effectively reduced the ATP percentage of the microflora on the cave surfaces. ATP measurement is a quantitative indicator of cell metabolism, commonly used in environmental microbiology to assess microbial activity in aquatic environments, oil slicks, caves, and to monitor bacterial contamination in food, pharmaceutical, and related industrial facilities [112]. Our findings are supported by other studies, demonstrating that EOs and their components such as carvacrol, eugenol, and cinnamaldehyde can reduce ATP production within bacterial cells [110,113,114]. For instance, oregano EO has been shown to effectively reduce intracellular ATP levels in *Staphylococcus aureus* [113]. Similar findings were reported by Oussalah et al. [108], who used ATP measurements to confirm the antibacterial activity of Spanish oregano and Chinese cinnamon EOs against *Escherichia coli* O157:H7 and *Listeria monocytogenes*. Our study demonstrates the antimicrobial action of EOs against the microbial mats found in the walls of the Petralona Cave. These results are in agreement with the research conducted by Argyri et al. [13], which showed the biocidal action of Origanum vulgare EO, among others, against microorganisms isolated from the Petralona cave walls. Although there are limited studies investigating the antimicrobial properties of EOs against microorganisms in cave environments, our findings are consistent with earlier research on the activity of EOs against various bacteria and fungi in different environments [104,107,115]. During this study, we used EOs in low concentrations, resulting in no strong odors being released into the cave environment and there was no adverse alteration of the color of the rocks during the application of the EOs.

## 4. Conclusions

The presence of contaminating microbial communities on cave walls and speleothems may become a significant concern, requiring effective treatment methods. Traditional cleaning approaches can spread the contamination or damage the cave structures, while chemical agents may also have negative effects on the cave’s environment. 16S and 18S rDNA amplicon sequencing has revealed the diverse range of microorganisms present on the walls and speleothems of the Petralona Cave, identifying various microbial genera and shedding light on the ecological complexity of cave ecosystems. Notably, the presence of some photosynthetic genera suggests their potential association with Lampenflora, which could be partly attributed to the artificial lighting installed inside the cave. To combat these unwanted microbial communities, we evaluated the antimicrobial efficacy of oregano and cinnamon EOs and their impact on ATP levels of the investigated biofilms. Our findings demonstrated that both essential oils drastically reduced the ATP levels during their application on all the spots tested. Subsequent spraying with a mixture solution of both EOs resulted in even greater reductions, reaching up to 96%. The utilization of oregano and cinnamon EOs and their effectiveness against the unwanted microbial mats found in the Petralona Cave showcases a novel and environmentally friendly approach to the treatment of microbiologically affected sites in caves. These results highlight the promising potential of EOs as alternative biocidal agents against the current methods which may be harmful for both the cave environments and the safety of visitors as well. Further research on the application of EOs in cave ecosystems is needed to fully understand their antimicrobial mechanisms and optimize their application for the control of unwanted microbes in caves.

## Figures and Tables

**Table 1:** Number of reads and OTUs for 16S rDNA and 18S rDNA amplicons of the three samples.

	16S rDNA Amplicon		18S rDNA Amplicon	
Samples	Number of Reads *	Number of OTUs	Number of Reads *	Number of OTUs
Green Spot 1	32,466	184	50,601	35
Green Spot 2	31,439	221	5761	35
Black Spot	24,411	189	44,977	59

* Numbers refer to reads assigned to OTUs at the genus level after quality control, removal of chimeric sequences and taxonomic assignment.

**Table 2:** Bioluminescence measurements in RLU units of speleothems before and after the application of essential oils of 0.15% oregano, 0.4% cinnamon, or their mixture, for 3 min (each).

AΤP (RLU)	Before EO Treatment	After Oregano Oil Treatment	After Cinnamon Oil Treatment	After Application of the Mixed Solution (Oregano and Cinnamon Oil)
Area 1 (green spot)	867.200	481.929 (44%) *	103.450 (88%)	45.163 (95%)
Area 2 (green spot)	148.226	20.561 (86%)	9.689 (93%)	5.719 (96%)
Area 3 (black spot)	299.099	70.206 (77%)	33.453 (89%)	16.230 (95%)
Mean % average	0 ± 0% ^a^	69.0 ± 22.1% ^b^	90.0 ± 2.6%^c^	95.3 ± 0.6% ^c^

* % reduction; letters in superscript indicate statistically homogeneous groups.

## Data Availability

Raw sequencing reads have been deposited in the sequence read archive (SRA) database, under access number SRR25020859 (16S rDNA Green_spot_1),SRR25021158 (16S rDNA Green_spot_2)SRR25021157 (16S rDNA Black_spot) and SRR25021516 (18S rDNA Green_spot_1),SRR25021515(18S rDNA Green_spot_2), SRR25021514(18S rDNA Black_spot). The rest of the data presented in this manuscript is available upon request.

## Supplementary Materials

The following supporting information can be downloaded at: https://www.mdpi.com/article/10.3390/microorganisms11112681/s1, Figure S1: Heatmap showing the relative abundances of the predicted enzyme sub-classes by PICRUSt2 across the three different sampling points based on 16S rDNA (A–F) or 18S rDNA (G–L).
