# Supplementary material for: 16S and 18S rDNA Amplicon Sequencing Analysis of Aesthetically Problematic Microbial Mats on the Walls of the Petralona Cave: The Use of Essential Oils as a Cleaning Method
Source: Microorganisms. 2023 Oct 31;11(11):2681. doi: 10.3390/microorganisms11112681 (PMC10673238; doi:10.3390/microorganisms11112681)
Supplement: Supplementary file 1 [file microorganisms-11-02681-s001.zip › microorganisms-2673350-supplementary.pptx]

## Slide 1
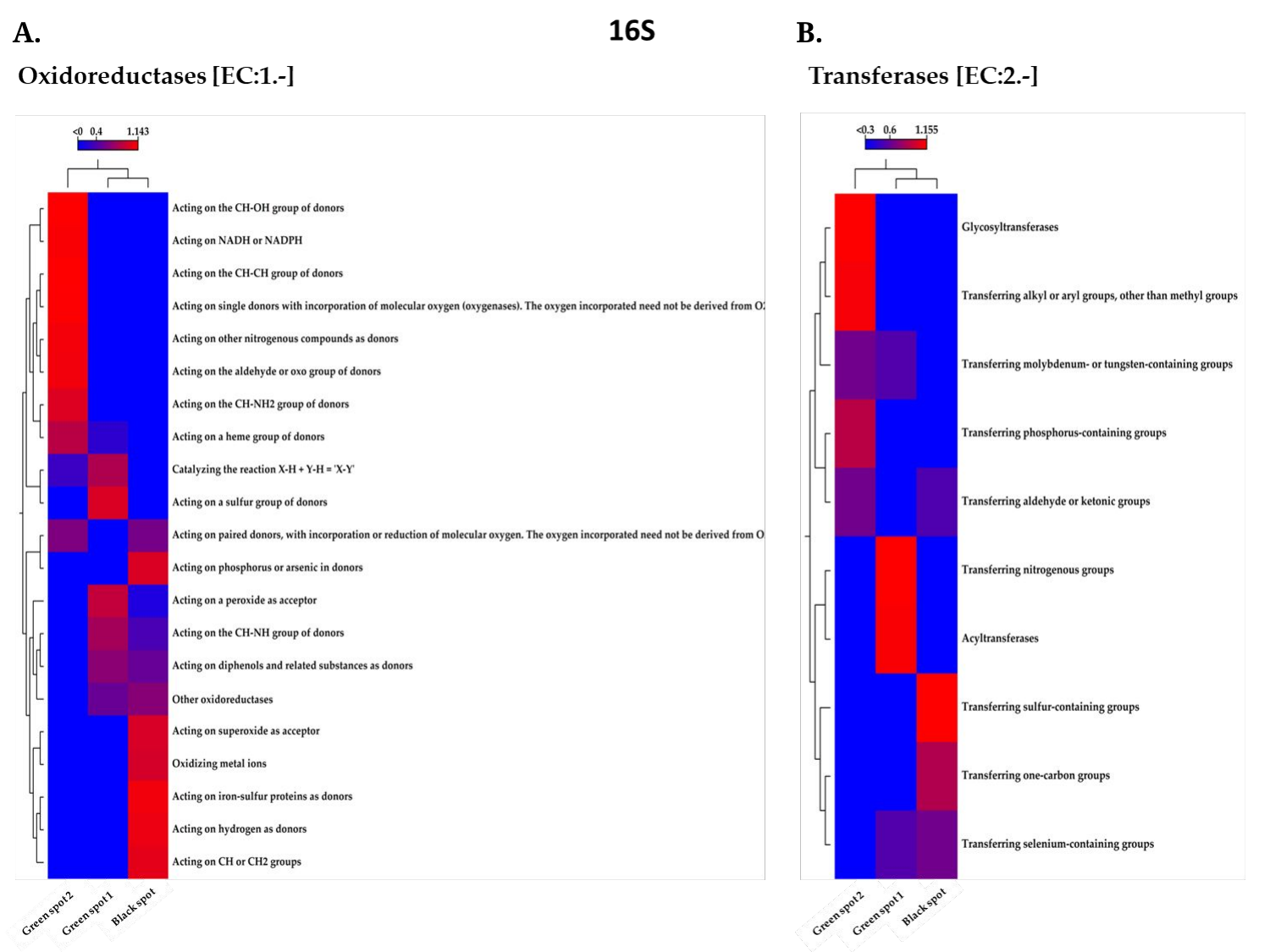

A.
B.

## Slide 2
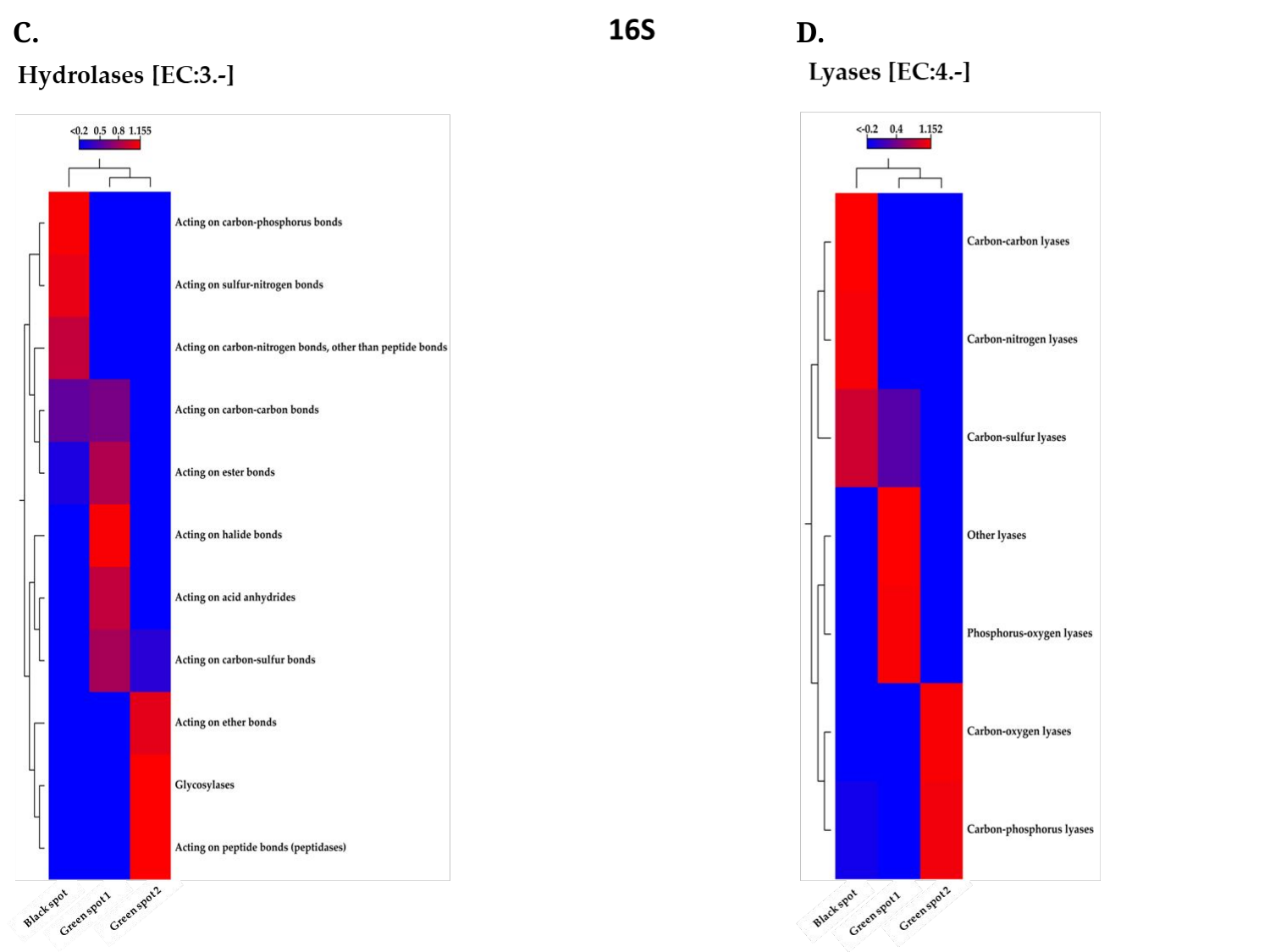

C.
D.

## Slide 3
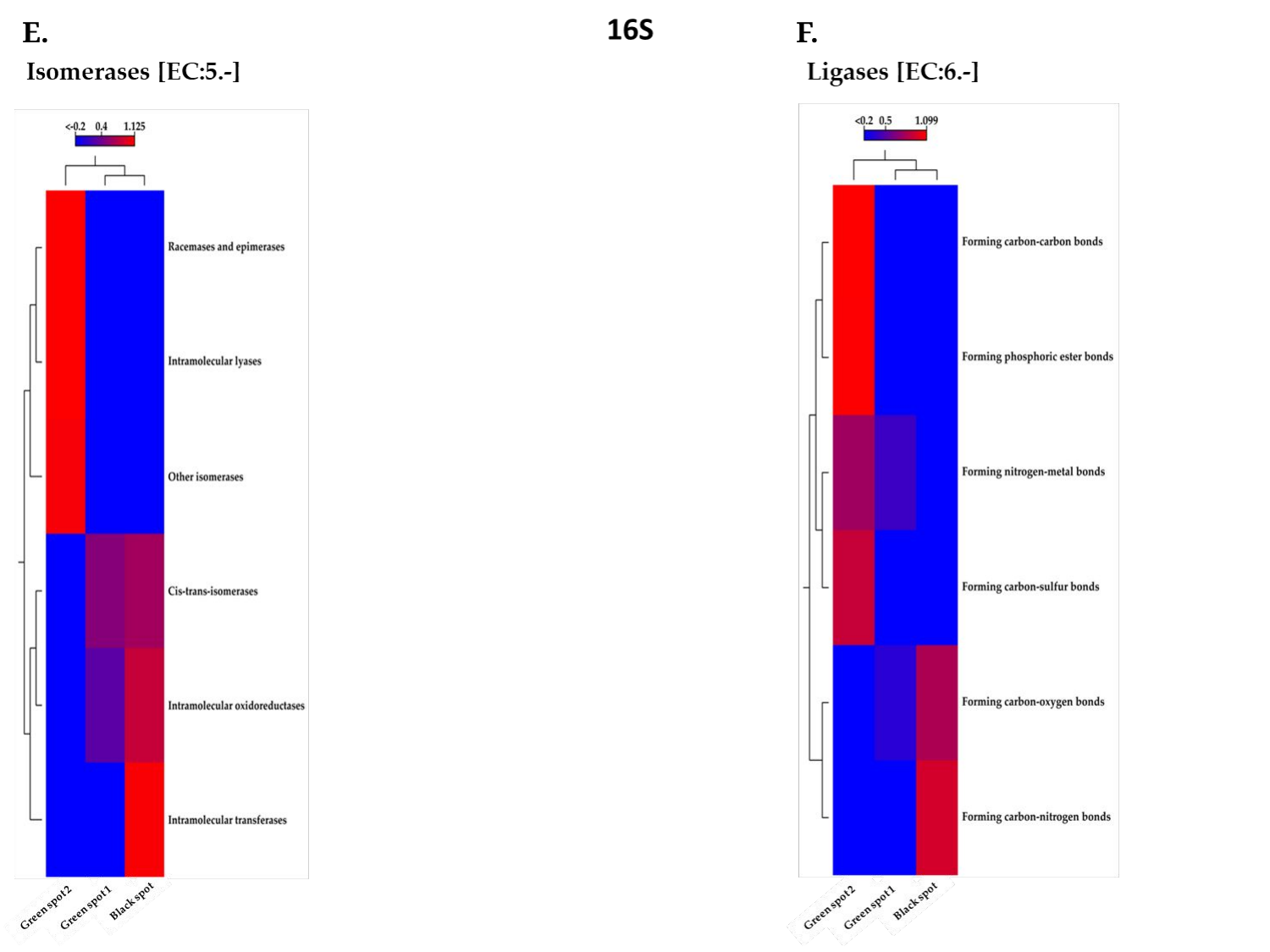

E.
F.

## Slide 4
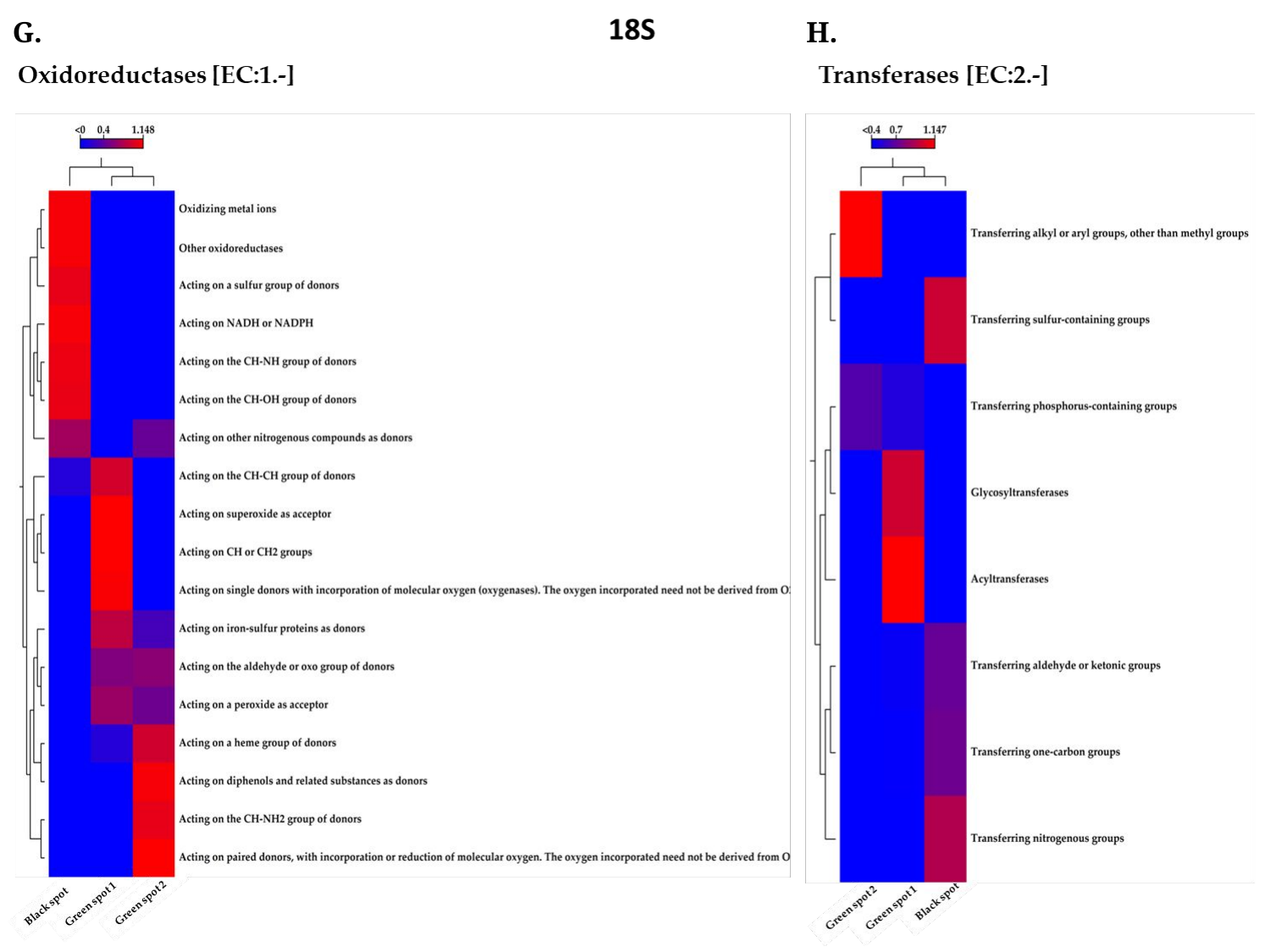

G.
H.

## Slide 5
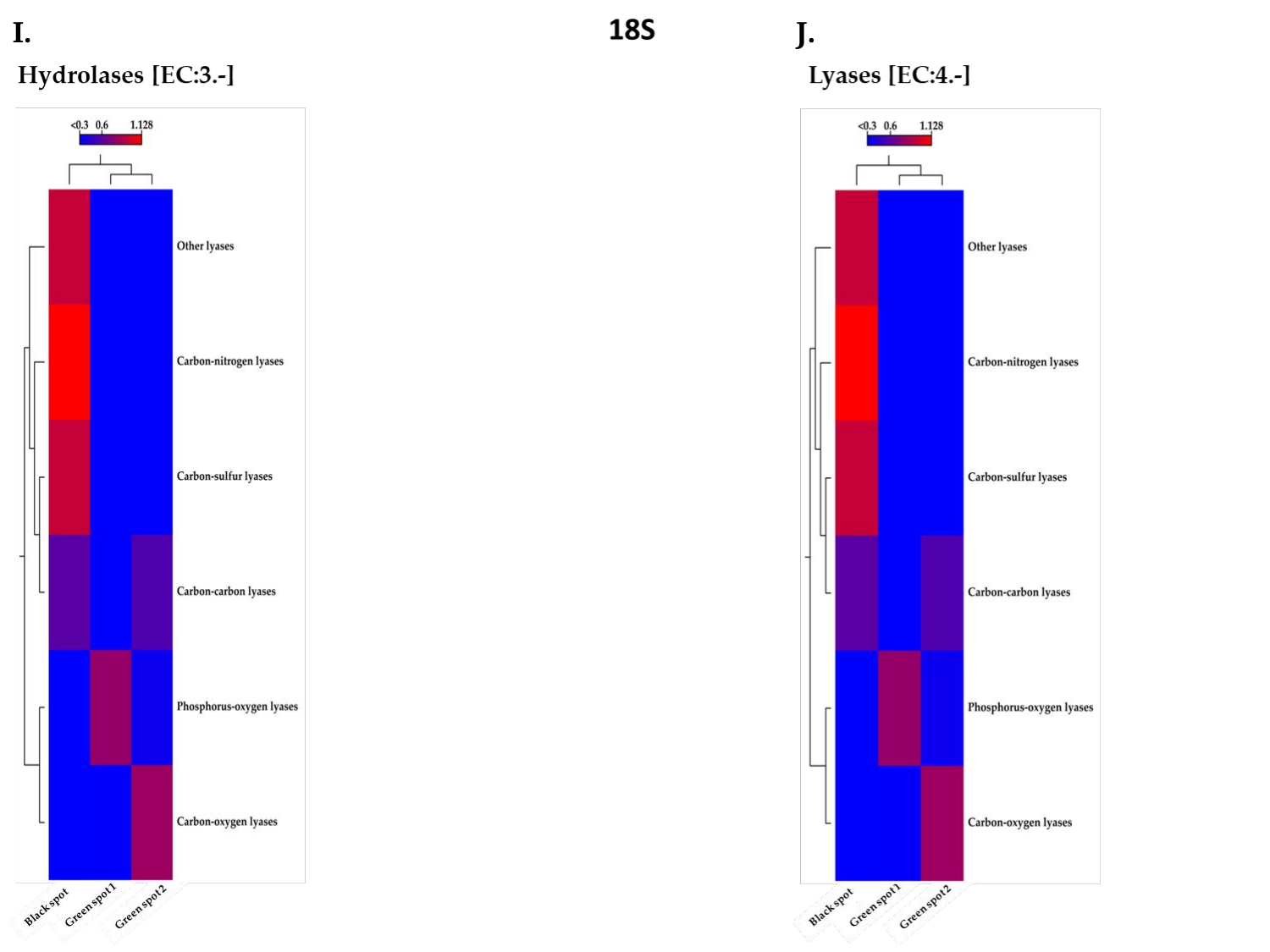

I.
J.

## Slide 6
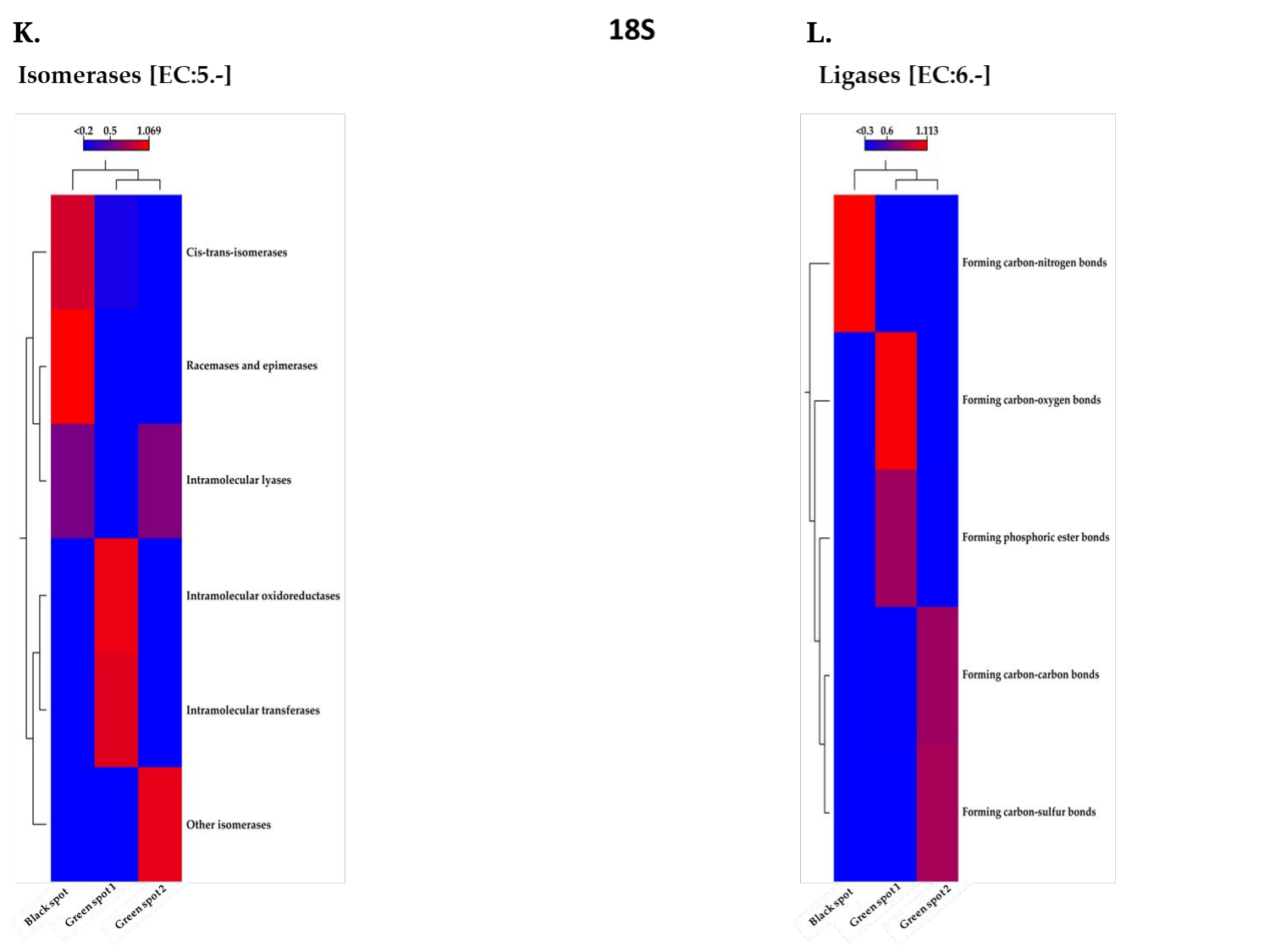

K.
L.

## Slide 7
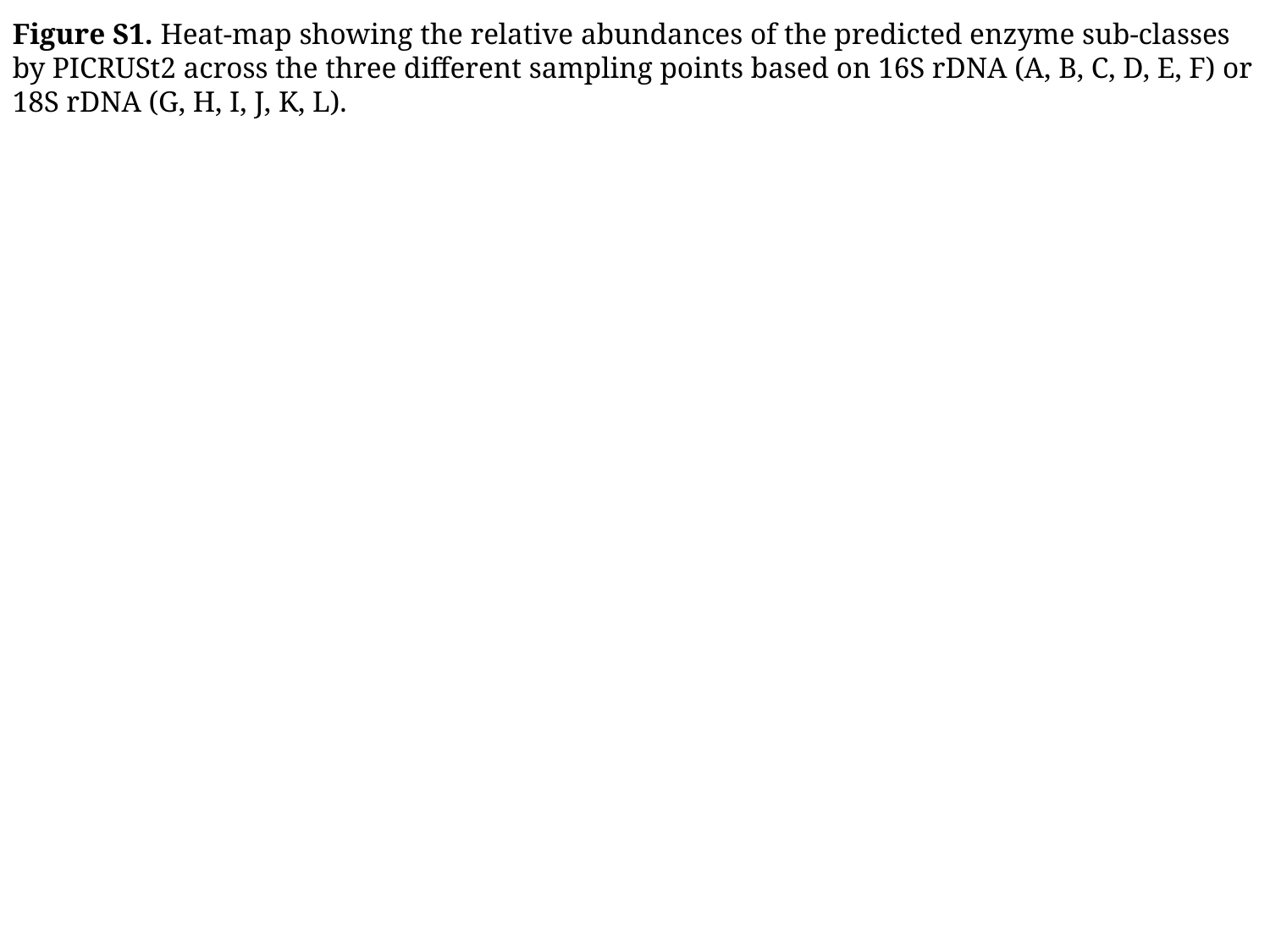

Figure S1. Heat-map showing the relative abundances of the predicted enzyme sub-classes by PICRUSt2 across the three different sampling points based on 16S rDNA (A, B, C, D, E, F) or 18S rDNA (G, H, I, J, K, L).
